# Association of arterial stiffness and heart failure with preserved ejection fraction in the elderly population – results from the CARLA study

**DOI:** 10.1038/s41371-022-00703-y

**Published:** 2022-05-17

**Authors:** Artjom Schott, Alexander Kluttig, Rafael Mikolajczyk, Karin Halina Greiser, Karl Werdan, Daniel Sedding, Sebastian Nuding

**Affiliations:** 1 Department of Internal Medicine III – Cardiology, Angiology and Internal Intensive Care Medicine, Mid-German Heart Center, University Hospital Halle (Saale), Halle (Saale), Germany; 2 Institute of Medical Epidemiology, Biostatistics, and Informatics, Interdisciplinary Center for Health Sciences, Medical Faculty of the Martin Luther University Halle-Wittenberg, Halle (Saale), Germany; 3 Division of Cancer Epidemiology, German Cancer Research Centre, Heidelberg, Germany

**Keywords:** Heart failure, Arterial stiffening

## Abstract

Arterial stiffness has been suspected as a cause of left ventricular diastolic dysfunction and may thereby contribute to the development of heart failure with preserved ejection fraction (HFpEF). However, this association is derived from a small number of studies and application of outdated criteria to diagnose HFpEF. This study aimed to investigate the association of arterial stiffness measured by the augmentation index (AIx) and criteria for diagnosing HFpEF according to the recommended HFA-PEFF score. Our analysis based on data from the first follow-up of the CARdiovascular Disease, Living and Ageing in Halle study. The current analysis included participants with available information about comorbidities and risk factors for HFpEF, parameters for calculation of the HFA-PEFF and noninvasive AIx estimated by applanation tonometry. The association of AIx and HFA-PEFF was investigated through descriptive and inductive statistics. A total of 767 participants were included in the analysis. AIx was associated with E/eʼ, left ventricular wall thickness (LVWT), relative wall thickness, left ventricular mass index (LVMI) and NT-proBNP but not with eʼ or left atrial volume index. However, after adjustment for confounders, only LVMI and LVWT remained associated with AIx. Males with a high AIx had a 3.2-fold higher likelihood of HFpEF than those with a low AIx. In contrast, that association was not present in females. In summary, AIx is associated with the morphological domain of the HFA-PEFF score represented by LVMI and LVWT. Higher values of AIx are associated with a higher likelihood for HFpEF in elderly males but not in females.

## Introduction

Heart failure with preserved ejection fraction (HFpEF) is a major global public health problem in the elderly population aged ≥60 years [1]. Currently, more than half of all heart failure hospital admissions in developed countries are caused by HFpEF [1]. This number is expected to increase given the increasing life expectancy. During the last decade, the understanding of the pathophysiology of HFpEF advanced, but it remains incomplete. The current paradigm of the development of HFpEF suggests that comorbidities, such as obesity, arterial hypertension, diabetes mellitus and chronic obstructive pulmonary disease induce a systemic inflammatory state, which causes coronary microvascular endothelial inflammation leading to reduced myocardial NO bioavailability [2, 3]. Subsequently, a deficient NO-cGMP-PKG signalling pathway may lead to concentric left ventricular (LV) remodelling, myocardial stiffness and diastolic dysfunction [2, 3]. Apart from that, based on the concept of ventricular-arterial coupling, arterial stiffness has been proposed to augment systolic blood pressure, increase cardiac afterload and decrease coronary perfusion with subendocardial ischaemia and fibrosis [4]. These adverse effects may contribute to the development of diastolic dysfunction and finally HFpEF [4]. Based on these pathophysiological considerations, arterial stiffness is supposed to be a key player in the development of HFpEF. Several parameters have been proposed to quantify arterial stiffness directly or indirectly. Aortic pulse wave velocity (PWV) is the gold standard to quantify arterial stiffness directly [5]. A true aortic PWV needs to be assessed invasively by catheters with pressure sensors above the aortic valve and aortic bifurcation [5]. Given the potential risks and technical requirements, ARTERY society guidelines proposed carotid-femoral PWV as the noninvasive reference standard, which can be estimated by pulse wave analysis using applanation tonometry [5]. However, in previous investigations, only brachial-ankle PWV and AIx showed an association with several parameters of diastolic dysfunction [6–9]. AIx was first defined in 1989 by Kelly et al. and describes the contribution of the reflected pressure wave to the central pressure wave, expressed as a dimensionless ratio or in percent [10]. Different devices applied at different arterial sites (radial, brachial, and carotid) can be used to estimate AIx [11, 12]. However, radial tonometry using the SphygmoCor system is proposed to be the best and most extensively validated technology, providing high within-observer and between-observer reproducibility [13, 14].

To date, several studies have investigated the association between arterial stiffness and echocardiographic indices of diastolic dysfunction [6–9, 15]. However, most of them were based on small studies and used outdated criteria to diagnose diastolic dysfunction and HFpEF. To overcome these limitations, our study aimed to investigate the association of arterial stiffness measured by AIx and the criteria for diagnosing HFpEF according to the current recommendations of the European Society of Cardiology (ESC) and Heart Failure Association (HFA) by applying a 3-domain-score (HFA-PEFF).

## Methods

Our analysis is based on data from the CARdiovascular Disease, Living and Ageing in Halle (CARLA) study. CARLA is an ongoing epidemiological prospective longitudinal cross-sectional cohort study of the elderly population of Halle (Saale), a city in Saxony-Anhalt (Germany), to investigate cardiovascular diseases in an ageing population and their association with behavioural, biomedical and psychosocial risk factors, including inflammation and heart rate variability. At the baseline examination, performed between 12/2002 and 01/2006, 1779 participants (812 women, 967 men) aged between 45 and 83 years were included in the study population. A 4-year follow-up could be accomplished in 1436 participants, while 343 persons died or refused to participate (participation rate 86%). During follow-up, all participating subjects underwent a detailed medical examination, including standardized transthoracic echocardiography, radial applanation tonometry, blood tests and a computer-assisted interview. A detailed description of the study design and methods have been published previously [16].

**Fig. 1:**
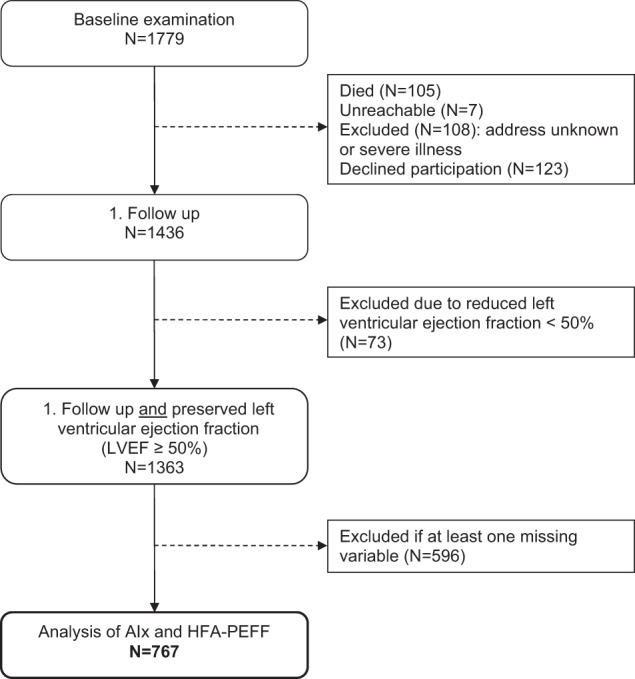
Flowchart illustrating the participants selection. AIx Augmentation index, LVEF left ventricular ejection fraction.

The study population was defined by the following inclusion criteria: available information (yes/no) about comorbidities of HFpEF (arterial hypertension ≥140/90 mmHg or use of antihypertensive drugs, atrial fibrillation according to Minnesota Code, diabetes mellitus or use of anti-diabetic drugs, sleep disorders with problems falling asleep), clinical signs for heart failure (dyspnoea and/or weakness at physical exercise, swollen legs in the evening, nightly urination), parameters describing potential risk factors (as obesity, lipid disorders, renal dysfunction, anaemia, inflammation, physical inactivity, smoking and alcohol consumption), and availability of parameters for the calculation of the HFA-PEFF score, including echocardiographic indices and NT-proBNP. Participants with a reduced left ventricular ejection fraction (LVEF) < 50% were excluded (*N* = 73). Furthermore, all participants with at least one missing value in one of the observed variables (which are presented in Tables 2 and 3) were excluded. Subsequently, 767 participants remained for analysis (Fig. 1).

The CARLA study was approved by the Ethics Committee of the Medical Faculty of Martin Luther University Halle-Wittenberg and conducted according to the principles of the Declaration of Helsinki [17]. All participants gave written informed consent.

**Fig. 2:**
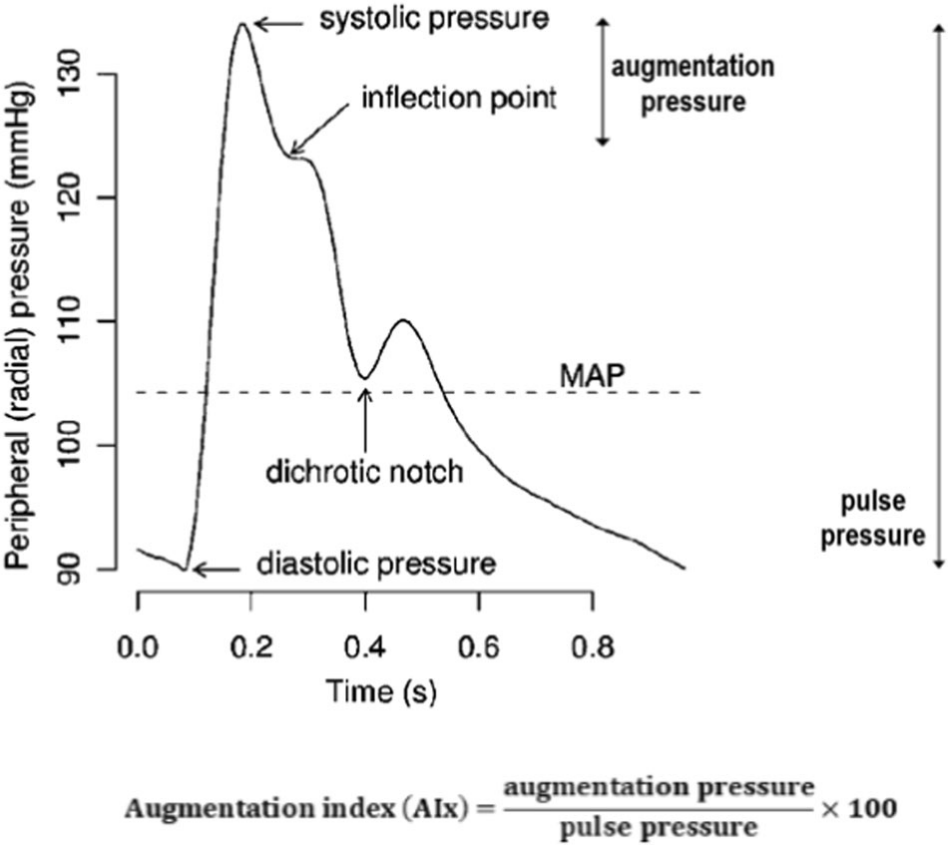
Illustration of a pressure pulse waveform. (Adapted from Alberto P Avolio et al. 2009 Physiol. Meas. 31 R1) MAP mean arterial pressure.

Arterial stiffness was estimated noninvasively by applanation tonometry of the radial artery using SphygmoCor (SphygmoCor, AtCor Medical, Sydney, Australia). Therefore, a pressure sensor was placed directly above the left or right radial artery in a sitting position after at least 10 min of rest. Pressure waveforms were recorded, and the key parameter AIx was used for analysis. AIx represents the augmentation of the central pressure wave by the peripheral reflected initial pressure wave (Fig. 2).

Standardized transthoracic echocardiography was performed by a trained investigator on a GE Vivid 5 (GE Medical Systems, Milwaukee, USA) and subsequently analysed by a trained physician. B-mode, M-mode, and Doppler mode examinations were performed in standardized parasternal and apical views. Septal e′, E/e′, left atrial volume index (LAVI), left ventricular mass index (LVMI), relative wall thickness (RWT) and left ventricular wall thickness (LVWT) were measured as specific echocardiographic parameters of the HFA-PEFF score. E/e′ was calculated using septal e′. LAVI was calculated based on atrial volume (measured in a four-chamber view) indexed to body surface area. Left ventricular mass was measured by the ASE convention and subsequently indexed to body surface area. RWT was calculated by the formula RWT = (2*posterior wall thickness)/LV diastolic diameter. LVWT was calculated by the formula LVWT = (diastolic interventricular septum + posterior wall thickness)/2.

The echocardiographic criteria were stratified according to the HFA-PEFF score (Table 1) [18]. The score consists of three different domains: functional, morphological and biomarker. Each domain is divided into major and minor criteria. A major criterion scores 2 points, and a minor criterion scores 1 point. Not all parameters of each domain needed to be obtained. The major and/or minor criteria within the same domain are not additive. Hence, every single domain can reach a maximum of 2 points. Points from different domains are summarized. A total score of ≥ 5 is considered to be diagnostic of HFpEF [18]. A total score of ≤1 is very unlikely to be associated with HFpEF. An intermediate total score from 2 to 4 points implied further evaluation (e.g., echo stress test, invasive haemodynamic measurements) is needed to clarify the diagnostic uncertainty. The participants of the present study were grouped into a low/intermediate (HFA-PEFF score <5) and high (HFA-PEFF score ≥5) HFA-PEFF group. To facilitate readability, participants in the high HFA-PEFF group are subsequently called HFpEF-probands.

**Table 1:** HFA-PEFF score according to the consensus recommendation from the Heart Failure Association (HFA) of the European Society of Cardiology (ESC).

	Functional		Morphological		Biomarker	
	Age <75 years	Age ≥75 years	SR	AF	SR	AF
Major	septal e′ < 7 cm/s *or* lateral e′ < 10 cm/s	septal e′ < 5 cm/s *or* lateral e′ < 7 cm/s	LAVI > 34 ml/m²	LAVI > 40 ml/m²	NT-proBNP > 220 pg/ml *or* BNP > 80 pg/ml	NT-proBNP > 660 pg/ml *or* BNP > 240 pg/ml
	*or* Average E/e′ ≥ 15 *or* TR velocity > 2,8 m/s (PASP > 35 mmHg)		*or* LVMI ≥ 149/122 g/m² (m/w) and RWT > 0,42			
Minor	Average E/e′ 9–14 *or* GLS < 16%		LAVI > 29–34 ml/m²	LAVI > 34–40 ml/m²	NT-proBNP 125–220 pg/ml *or* BNP 35–80 pg/ml	NT-proBNP 375–660 pg/ml *or* BNP 105–240 pg/ml
			*or* LVMI ≥ 115/95 g/m² (m/w) *or* RWT > 0,42 *or* LVWT ≥ 12 mm			

*AF* atrial fibrillation, *BNP* brain natriuretic peptide, *e*′ mitral annular peak early diastolic velocity, *E/e′* ratio of mitral inflow and mitral annular peak early diastolic velocity, *GLS* global longitudinal strain, *LAVI* left atrial volume index, *LVMI* left ventricular mass index, *LVWT* left ventricular wall thickness, *m/w* men/women, *NT-proBNP* N-terminal prohormone brain natriuretic peptide, *PASP* pulmonary arterial systolic pressure, *RWT* relative wall thickness, *SR* sinus rhythm, *TR* tricuspid regurgitation.

Descriptive statistics of patient characteristics and parameters of the HFA-PEFF score are presented as the mean ± standard deviation with absolute numbers and relative percentages. The correlation was analysed by Spearman correlation. Univariate linear regression analysis was used to account for potential confounders. z-standardized estimates were used to account for various unit sizes. Binary logistic regression analysis was used to investigate the association of AIx and HFA-PEFF groups.

Statistical analysis was performed by SPSS (IBM Corp. Released 2016. IBM SPSS Statistics for Windows, Version 24.0. Armonk, NY: IBM Corp.).

## Results

Overall, 767 elderly participants (354 females and 413 males) aged between 50 and 87 years were included in the descriptive analysis (Table 2). HFpEF-probands more often showed typical comorbidities and risk factors for HFpEFs, such as advanced age, arterial hypertension, atrial fibrillation, diabetes mellitus, renal dysfunction, sleep disorders and elevated NT-proBNP, than participants with low or intermediate HFA-PEFF scores. Similar findings were observed for clinical signs of heart failure. HFpEF-probands more often reported dyspnoea and/or weakness at physical exercise, swollen legs in the evening and nightly urination compared to lower HFA-PEFF scores.

**Table 2:** Participant characteristics including risk factors, comorbidities and clinical signs for heart failure presented by mean ± SD and count (*N* %).

		All participants (*N* = 767)	Low or Intermediate HFA-PEFF (*N* = 652)	High HFA-PEFF (*N* = 115)
Sex [male]		413 (53.8%)	353 (54.1%)	60 (52.2%)
Sex [female]		354 (46.2%)	299 (45.9%)	55 (47.8%)
Age [years]		65.36 ± 9.06	64.17 ± 8.73	72.10 ± 7.88
Body height [m]		1.67 ± 0.09	1.68 ± 0.09	1.65 ± 0.09
BMI [kg/m²]		28.00 ± 4.25	27.83 ± 4.20	29.00 ± 4.38
HR [min^-1^]		67.78 ± 10.35	68.42 ± 10.13	64.17 ± 10.89
SBP [mmHg]		136.76 ± 19.17	135.90 ± 18.69	141.67 ± 21.13
DBP [mmHg]		80.69 ± 9.89	81.04 ± 9.56	78.74 ± 11.47
MAP [mmHg]		99.38 ± 11.79	99.32 ± 11.51	99.71 ± 13.31
Cholesterol [mmol/l]		5.49 ± 1.02	5.54 ± 1.03	5.21 ± 0.97
HDL [mmol/l]		1.35 ± 0.38	1.37 ± 0.39	1.25 ± 0.32
LDL [mmol/l]		3.38 ± 0.90	3.41 ± 0.89	3.19 ± 0.91
Triglyceride [mmol/l]		1.87 ± 1.05	1.86 ± 1.03	1.93±1.15
NT-proBNP [pg/ml]		175.01 ± 261.91	125.32 ± 195.43	456.72 ± 385.53
Creatinine [µmol/l]		78.65 ± 28.98	76.09 ± 20.80	93.18 ± 54.06
Hb [g/dl]		16.46 ± 2.78	16.47 ± 2.70	16.42 ± 3.23
HbA1c [%]		5.83 ± 0.70	5.79 ± 0.68	6.03 ± 0.75
hsCRP [mg/l]		3.29 ± 7.40	2.88 ± 5.02	5.63 ± 14.75
Physical activity [h/week]		1.61 ± 2.52	1.58 ± 2.47	1.81 ± 2.78
Arterial hypertension or antihypertensive drugs	No	208 (27.1%)	198 (30.4%)	10 (8.7%)
	Yes	559 (72.9%)	454 (69.6%)	105 (91.3%)
Atrial fibrillation (according to Minnesota Code)	No	747 (97.4%)	641 (98.3%)	106 (92.2%)
	Yes	20 (2.6%)	11 (1.7%)	9 (7.8%)
Prior myocardial infarction	No	728 (94.9%)	630 (96.6%)	98 (85.2%)
	Yes	39 (5.1%)	22 (3.4%)	17 (14.8%)
Diabetes mellitus or antidiabetic drugs	No	656 (85.5%)	576 (88.3%)	80 (69.6%)
	Yes	111 (14.5%)	76 (11.7%)	35 (30.4%)
Chest pain or discomfort	No	565 (73.7%)	493 (75.6%)	72 (62.6%)
	Yes	202 (26.3%)	159 (24.4%)	43 (37.4%)
Dyspnea or weakness at physical exercise	None	461 (60.1%)	415 (63.7%)	46 (40.0%)
	Only dyspnea	180 (23.5%)	136 (20.9%)	44 (38.3%)
	Only weakness	48 (6.3%)	40 (6.1%)	8 (7.0%)
	Both	78 (10.2%)	61 (9.4%)	17 (14.8%)
Swollen legs at evening	No	616 (80.3%)	533 (81.7%)	83 (72.2%)
	Yes	151 (19.7%)	119 (18.3%)	32 (27.8%)
Urination at night	No	339 (44.2%)	314 (48.2%)	25 (21.7%)
	Yes	428 (55.8%)	338 (51.8%)	90 (78.3%)
Problems falling asleep	Never	268 (34.9%)	230 (35.3%)	38 (33.0%)
	Sometimes	383 (49.9%)	333 (51.1%)	50 (43.5%)
	Often	116 (15.1%)	89 (13.7%)	27 (23.5%)
Problems staying asleep	Never	142 (18.5%)	127 (19.5%)	15 (13.0%)
	Sometimes	395 (51.5%)	344 (52.8%)	51 (44.3%)
	Often	230 (30.0%)	181 (27.8%)	49 (42.6%)
Smoking	Never	341 (44.5%)	291 (44.6%)	50 (43.5%)
	Ex	296 (38.6%)	243 (37.3%)	53 (46.1%)
	Occasional	13 (1.7%)	13 (2.0%)	0 (0.0%)
	Current	117 (15.3%)	105 (16.1%)	12 (10.4%)
Alcohol [l/week]		0.09 ± 0.13	0.10 ± 0.13	0.09 ± 0.12

*BMI* body mass index, *DBP* diastolic blood pressure, *Hb* haemoglobin, *HbA1c* gylcosylated haemoglobin, *HDL* high density lipoprotein, *HR* heart rate, *hsCRP* high sensitivity C-reactive protein, *LDL* low density lipoprotein, *MAP* mean arterial pressure, *NT-proBNP* N-terminal prohormone brain natriuretic peptide, *SBP* systolic blood pressure.

Parameters of AIx and HFA-PEFF score are presented in Table 3 organized according to their groups. Among all participants, AIx did not differ between the HFA-PEFF groups. However, subgroup analysis showed sex-related differences. Females had higher AIx than males independent of the HFA-PEFF score (33.57% vs. 25.91%; −7.67 [−9.02; −6.32] (difference [95% CI])), also when observed separately in HFpEF-probands (33.29% vs. 29.00%; −4.29 [−7.85; −0.73]) and lower HFA-PEFF scores (33.63% vs. 25.38%; −8.24 [−9.70; −6.79]). Within female participants, AIx did not differ between HFpEF-probands and those with lower HFA-PEFF scores (33.29% vs. 33.63%, 0.34 [−2.38; 3.05]). In contrast, male HFpEF-probands had a higher AIx than those with lower HFA-PEFF scores (29.00% vs. 25.38%, −3.62 [−6.23; −1.00]).

**Table 3:** Measurements of AIx, LVEF and parameters of HFA-PEFF Score categorized by HFA-PEFF group presented by difference and 95% confidence interval [95% CI].

	Low or Intermediate HFA-PEFF (*N* = 652)	High HFA-PEFF (*N* = 115)	Difference [95% CI]
AIx [%]	29.16 ± 10.30	31.05 ± 9.82	−1.89 [−3.92; 0.14]
LVEF [%]	61.54 ± 5.80	61.41 ± 5.13	0.13 [−1.00; 1.27]
eʼ [cm/s]	6.55 ± 1.81	5.37 ± 1.42	1.19 [0.89; 1.48]
E/eʼ [ratio]	10.74 ± 3.41	14.25 ± 5.65	−3.51 [−4.58; −2.43]
LAVI [ml/m²]	20.34 ± 6.95	30.05 ± 12.74	−9.71 [−12.12; −7.29]
LVMI [g/m²]	104.69 ± 31.69	129.03 ± 31.69	−24.34 [−30.49; −18.20]
RWT [ratio]	0.45 ± 0.10	0.48 ± 0.11	−0.03 [−0.05; −0.01]
LVWT [mm]	10.92 ± 1.68	11.91 ± 1.65	−0.99 [−1.32; −0.66]
NT−proBNP [pg/ml]	125.32 ± 195.43	456.72 ± 385.52	−331.40 [−404.15; −258.65]

*AIx* Augmentation index, *e*′ mitral annular peak early diastolic velocity, *E/e*′ ratio of mitral inflow and mitral annular peak early diastolic velocity, *LAVI* left atrial volume index, *LVEF* left ventricular ejection fraction, *LVMI* left ventricular mass index, *LVWT* left ventricular wall thickness, *NT-proBNP* N-terminal prohormone brain natriuretic peptide, *RWT* relative wall thickness.

Left ventricular ejection fraction (LVEF) did not differ significantly between the HFA-PEFF groups. In contrast, functional (e′, E/e′), morphological (LAVI, LVMI, RWT, and LVWT) and biomarker (NT-proBNP) HFA-PEFF indices were more pathologically unstable in HFpEF-probands.

**Fig. 3:**
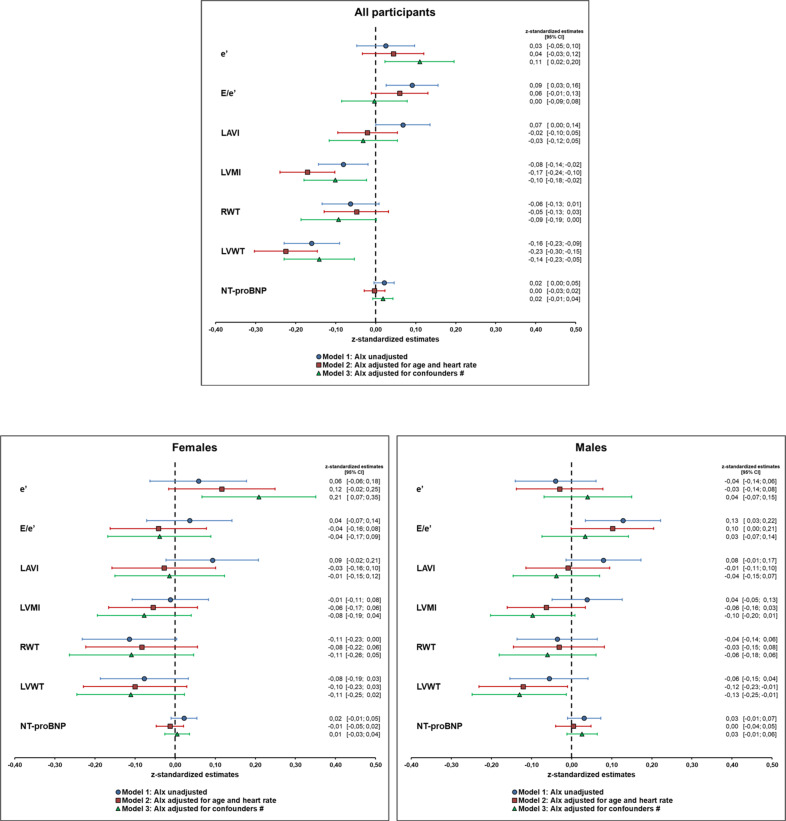
Illustration of the linear regression models presented by forest plot for all participants, females and males. The association of AIx and parameters of the HFA-PEFF score is presented by z-standardized estimates. AIx was analysed unadjusted (Model 1) as well as adjusted for confounders (Model 2 and 3). e′ mitral annular peak early diastolic velocity, E/e′ ratio of mitral inflow and mitral annular peak early diastolic velocity, LAVI left atrial volume index, LVMI left ventricular mass index, RWT relative wall thickness, LVWT left ventricular wall thickness, NT-proBNP N-terminal prohormone brain natriuretic peptide.

**Fig. 4:**
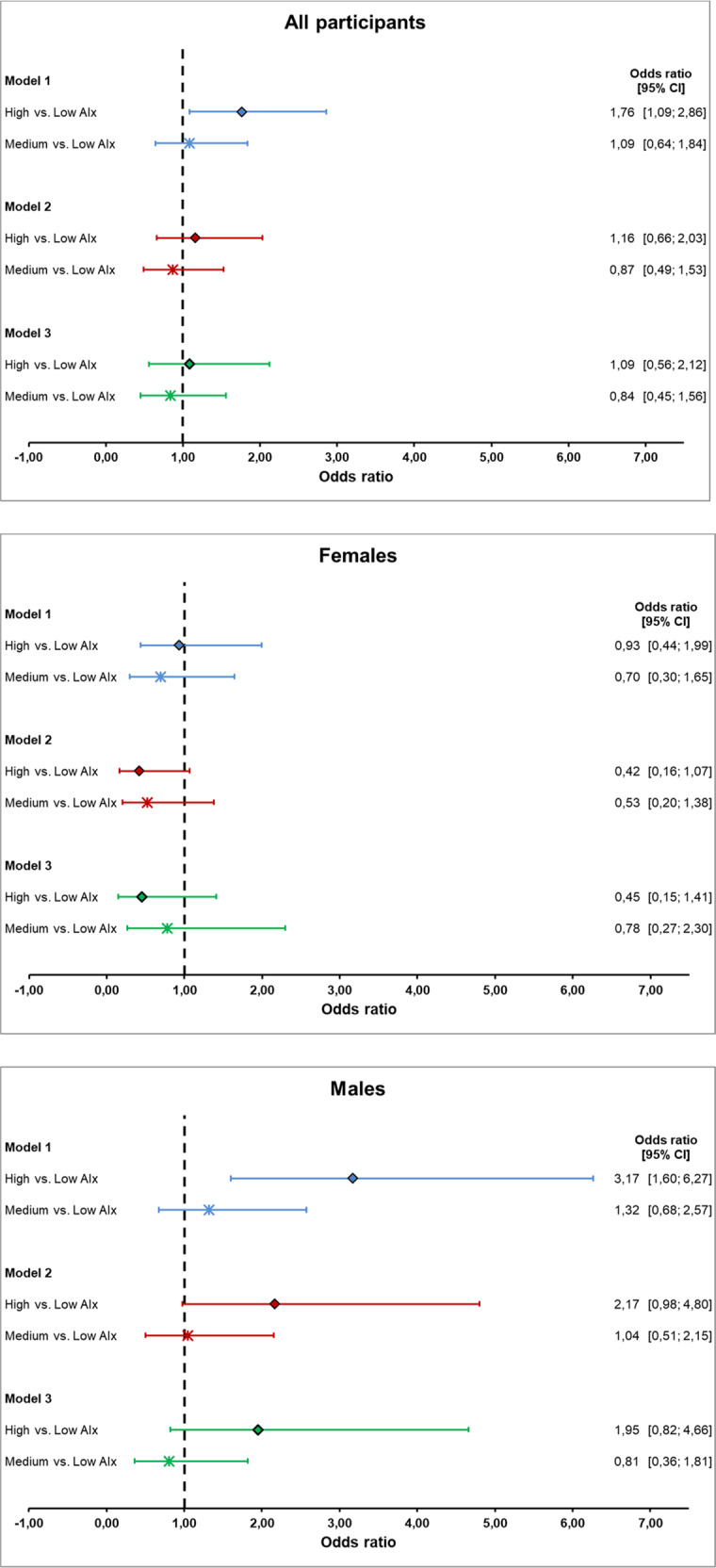
Illustration of the binary logistic regression models presented by forest plot for all participants, females and males. The association of AIx and HFA-PEFF score is presented by Odds ratio. AIx was analysed unadjusted (Model 1) as well as adjusted for confounders (Model 2 and 3). AIx was grouped into tertiles defined as high, medium and low (the low tertile serves as the reference category). The HFA-PEFF score was grouped binary (low/intermediate and high).

AIx correlated positively with E/é and NT-proBNP and inversely with LVWT, RWT and LVMI but not with é and LAVI. When performing linear regression analysis, AIx was influenced by E/e′, LAVI, LVMI and LVWT but not by e′, RWT or NT-proBNP. However, after adjustment for confounders (age, sex, body height, heart rate, mean arterial pressure, antihypertensive drugs, left ventricular ejection fraction, atrial fibrillation, diabetes mellitus, cholesterol, creatinine and C-reactive protein), only LVMI and LVWT remained associated with AIx (Fig. 3). Stratification by gender did not present relevant differences for those adjusted effect sizes. AIx (unadjusted) was associated with a slightly higher probability for HFpEF in males but not in females (Table 4). Male participants with a high AIx (i.e., the AIx value in the highest tertile of the study population) presented a 3.2-fold higher likelihood for HFpEF compared to those with a low AIx (i.e., AIx value in the lowest tertile of the study population). However, those associations weakened after adjustment for the confounders mentioned above (Fig. 4).

**Table 4:** Binary logistic regression models for AIx and binary grouped HFA-PEFF (low/intermediate vs. high) presented by Odds ratio (OR) of HFA-PEFF per unit increase of AIx and 95% confidence interval [95% CI].

	All participants (*N* = 767)	Female (*N* = 354)	Male (*N* = 413)
Model 1: AIx unadjusted	1.02 [1.00; 1.04]	1.00 [0.97; 1.03]	1.04 [1.01; 1.07]
Model 2: AIx adjusted for age and heart rate	1.00 [0.98; 1.02]	0.95 [0.91; 0.99]	1.02 [0.99; 1.06]
Model 3: AIx adjusted for confounders^a^	0.99 [0.97; 1.02]	0.95 [0.90; 1.00]	1.02 [0.98; 1.06]

^a^Age, sex, body height, heart rate, mean arterial pressure, antihypertensive drugs, left ventricular ejection fraction, atrial fibrillation, diabetes mellitus, cholesterol, creatinine and C-reactive protein.

## Discussion

Arterial stiffness is supposed to be a key player in the development of HFpEF. Our study demonstrated an association of arterial stiffness measured by AIx and HFpEF according to the HFA-PEFF score. AIx correlates with several domains of the HFA-PEFF score. However, after adjustment for confounders, LVMI and LVWT remained the only items of the HFA-PEFF score to be associated with AIx. Subgroup analyses revealed sex-related differences. An increase in AIx is associated with a higher probability for HFpEF in males but not in females. In particular, males with a high AIx present a higher risk for HFpEF than those with a low AIx. However, that association was weakened after adjustment for confounders.

Several participant characteristics are potential confounders of arterial stiffness and thus were considered in our analysis. In particular, age- and sex-related differences in AIx have been well investigated [9, 10, 19–21]. Ageing is accompanied by structural changes in the arterial system (i.e., degeneration of elastin, increase in collagen, and thickening of the arterial wall), leading to increased arterial stiffness [22]. According to previous investigations, the age-related increase in AIx seems to be nonlinear and flattens in elderly individuals older 60 years of age [19, 20]. The reason for this curvilinear relationship remains uncertain. Mitchell et al. hypothesized a higher age-related increase in central aortic stiffness compared to peripheral arterial stiffness leading to reduced and distally shifted wave reflections resulting in lower AIx [20]. In contrast, Namasivayam et al. proposed the curvilinear relationship of AIx and age to be a mathematical phenomenon as both components of the AIx, i.e., augmentation pressure and pulse pressure, were reported to have a positive linear relationship with age [23]. Our study does not identify age-related differences in AIx, most likely as two-thirds of our population were aged > 60 years. In contrast, our study confirms sex-related differences in AIx. Women have higher AIx than men, as reported in prior investigations [9, 21, 24].

Multiple further confounders, such as body height, heart rate, mean arterial pressure, antihypertensive drugs, left ventricular ejection fraction, atrial fibrillation, diabetes mellitus, cholesterol, creatinine and C-reactive protein, have been reported by previous investigators to potentially modify AIx and thus have been considered in our regression analysis [25–32]. The adjustment for these confounders reveals LVWT as the strongest HFA-PEFF item to be influenced by arterial stiffness.

In addition to antihypertensives, other groups of drugs have been reported to be potential confounders of arterial stiffness. For instance, statins are supposed to act anti-inflammatory and anti-oxidative at the arterial wall and may thereby lead to improved arterial stiffness [33]. Similarly, anti-diabetics such as glitazones and metformin have been reported to decrease arterial stiffness [33]. Finally, corticosteroids and acetylsalicylic acid are also supposed to improve arterial stiffness due to their anti-inflammatory effect [33]. However, previous investigations of those other drugs provided heterogeneous results. Hence, we focused on antihypertensives, which provide the most evidence to act as confounders of arterial stiffness [33].

During the last decade, the criteria for diastolic dysfunction and HFpEF have been changed. One of the recent updates was the introduction of the HFA-PEFF score to facilitate the challenging diagnostic algorithm for suspected HFpEF [18]. To our knowledge, our study is the first analysis focusing on the association of AIx and HFpEF considering the recommended HFA-PEFF score. In contrast to previous investigations, our study was not confined to echocardiographic criteria of diastolic dysfunction (e′, E/e′) but also considered morphological criteria (LAVI, LVMI, RWT, LVWT) and the biomarker domain represented by NT-proBNP. Previous studies focused on echocardiographic indices of diastolic dysfunction, such as E/A, e′ and E/e′, to estimate HFpEF [6, 8, 9]. However, none of these indices can reliably confirm the diagnosis of HFpEF. In particular, changes in E/A are age-dependent and may appear similar across different stages of diastolic dysfunction. Indeed, only eʼ and E/eʼ but not E/A are included in the calculation of the HFA-PEFF score. Hence, our study did not investigate the association of AIx and E/A.

E/eʼ is an established parameter for the noninvasive estimation of left ventricular filling pressure [34]. However, in certain clinical conditions (e.g., moderate to severe mitral regurgitation or stenosis, severe mitral annular calcification, left bundle branch block, cardiac resynchronization therapy), E/eʼ may be less accurate and should be interpreted with caution [34]. These clinical conditions could have potentially influenced the measurements of E/eʼ in our CARLA study. However, other functional, morphological and biomarker criteria for HFpEF were considered in our analysis.

Previous investigations reported an association of AIx with different parameters of diastolic dysfunction, including E/A, e′ and E/e′ [6–9, 15]. These results are not entirely confirmed by our study. AIx correlated with E/e′ but not with e′. However, this association weakens after adjustment for our reported confounders. These differing results may have multiple reasons, as previous studies investigated a younger or disease-specific population (e.g., individuals with hypertension, atrial fibrillation or diabetes mellitus) [8, 9, 15, 35], used other techniques (e.g., cardiac catheterization, automated tonometry system) or arterial sites to measure AIx [7, 35] and used other thresholds for parameters of diastolic dysfunction according to outdated guidelines [7–9, 15, 35].

In contrast to diastolic dysfunction, parameters of left ventricular dimension and mass showed a marked association with AIx. According to our study, AIx is negatively correlated with LVMI and LVWT. At first glance, these results seem to be unexpected, but similar observations have been reported in previous studies and are pathophysiologically explainable [36, 37]. In radially recorded arterial pressure waves, the age-related increase in arterial stiffness is mainly represented by an increase in pulse pressure instead of the augmentation pressure [10]. Hence, the ratio of augmentation pressure and pulse pressure, which represents the AIx, decreases. Simultaneously, central blood pressure increases with age, leading to increased cardiac afterload and LV hypertrophy [4]. Thus, our inverse association of AIx and parameters of LV hypertrophy becomes conclusive.

To our knowledge, the association of AIx and the HFA-PEFF score has not yet been reported. Our study identifies a higher probability for HFpEF, especially in males with a high AIx but not in females. Notably, our results are observed in a general elderly population. Hospitalized patients with higher morbidity most likely have higher HFA-PEFF scores and thereby a possibly differing association with AIx than those with lower morbidity. Further research is needed to clarify this uncertainty.

Our results are based on data from the CARLA study, which was designed before the introduction of the HFA-PEFF score. Lateral e′, tricuspid regurgitation velocity and global longitudinal strain were not obtained and could thus not be included in our calculation of the HFA-PEFF score. Nevertheless, the HFA-PEFF score can be calculated even if not all parameters are available [18]. Furthermore, in individuals with an intermediate HFA-PEFF score, further evaluation is recommended to confirm or exclude the diagnosis of HFpEF. These examinations were not performed in our study. Whether this missing evaluation could have influenced our results remains uncertain.

In summary, AIx is associated with the morphological domain of the HFA-PEFF score represented by LVMI and LVWT. Higher values of AIx are associated with a higher likelihood for HFpEF in elderly males but not in elderly females.

### Summary table

#### What is known about topic

- Arterial stiffness has been proposed to contribute to the development of left ventricular diastolic dysfunction and heart failure with preserved ejection fraction (HFpEF).
- Small studies reported an association of arterial stiffness measured by augmentation index (AIx) and meanwhile outdated criteria to diagnose HFpEF.

#### What this study adds

- Our study investigated the association of arterial stiffness measured by augmentation index (AIx) and criteria for diagnosing HFpEF according to the currently recommended HFA-PEFF score in a large sampled study population.
- AIx is associated with the morphological domain of the HFA-PEFF score represented by left ventricular mass index (LVMI) and left ventricular wall thickness (LVWT).
- Higher values of AIx are associated with a higher likelihood for HFpEF in elderly males but not in females.

## Data Availability

Data may be requested from the CARLA study steering committee in accordance with the data use regulations.
